# J3Gen: A PRNG for Low-Cost Passive RFID

**DOI:** 10.3390/s130303816

**Published:** 2013-03-19

**Authors:** Joan Melià-Seguí, Joaquin Garcia-Alfaro, Jordi Herrera-Joancomartí

**Affiliations:** 1 Department of Communications and Information Technologies, Universitat Pompeu Fabra, Tanger 122-140, 08018 Barcelona, Spain; 2 Internet Interdisciplinary Institute, Universitat Oberta de Catalunya, Roc Boronat 117, 08018 Barcelona, Spain; E-Mails: joaquin.garcia-alfaro@acm.org (J.G.-A.); jordi.herrera@uab.cat (J.H.-J.); 3 RST Department, Institut Mines-Telecom, Telecom SudParis, CNRS Samovar UMR 5157, 9 Rue Charles Fourier, 91000 Evry, France; 4 Department of Information and Communications Engineering, Universitat Autònoma de Barcelona, Edifici Q, Campus de Bellaterra, 08193 Bellaterra, Spain

**Keywords:** PRNG, security, LFSR, multiple-polynomial, RFID, EPC Gen2

## Abstract

Pseudorandom number generation (PRNG) is the main security tool in low-cost passive radio-frequency identification (RFID) technologies, such as EPC Gen2. We present a lightweight PRNG design for low-cost passive RFID tags, named J3Gen. J3Gen is based on a linear feedback shift register (LFSR) configured with multiple feedback polynomials. The polynomials are alternated during the generation of sequences via a physical source of randomness. J3Gen successfully handles the inherent linearity of LFSR based PRNGs and satisfies the statistical requirements imposed by the EPC Gen2 standard. A hardware implementation of J3Gen is presented and evaluated with regard to different design parameters, defining the key-equivalence security and nonlinearity of the design. The results of a SPICE simulation confirm the power-consumption suitability of the proposal.

## 1. Introduction

The Electronic Product Code Class 1 Generation 2 [1] (EPC Gen2 for short) is a passive low-cost radio-frequency identification (RFID) technology for automated identification over Ultra High Frequency (UHF) interfaces. EPC Gen2 compliant RFID tags are passive electronic labels powered by the electromagnetic field of RFID readers, with a typical reading distance of up to five meters. The main constraints to integrate security features on-board of EPC Gen2 tags are power consumption, performance and compatibility requirements, which can be summarized in the cost of the security features. EPC Gen2 tags only consider two main security elements: a 16-bit pseudorandom number generator (PRNG) and password-protected operations (using the PRNG as a cipher tool). The PRNG is also used as an anti-collision mechanism for inventorying processes and to acknowledge other EPC Gen2 specific operations. The on-board 16-bit PRNG is, therefore, the crucial component that guarantees the security of a Gen2 tag.

EPC Gen2 manufacturers do not provide their PRNG designs [2]. They refer to testbeds demonstrating the accomplishment of the requirements defined in the EPC Gen2 standard for PRNG generation [1], failing to offer convincing information about the security of their designs [3]. This is mostly security through obscurity, which is always ineffective in security engineering, as it has been shown with the disclosure of the PRNG used in the MIFARE Classic chip [4] that has shown a vulnerable PRNG. The use of weak PRNGs that allow the predictability of the outgoing sequences also introduces important security flaws in EPC Gen2 communications. For example, it might allow an adversary to bypass the security of the password-protected commands defined in the Gen2 standard [3].

In this paper, we present a lightweight PRNG scheme for EPC Gen2 tags named J3Gen, which is based on a preliminary design presented in [5] by the same authors. First of all, we describe the system design and the system components. Later on, we analyze the proposed scheme in terms of security, studying how some design parameters can act as a security key and which level of security can be obtained depending on the parameters set up. Then, we present an analysis of the suitability of the different parameters that can be set up in our scheme. We propose a specific configuration that offers the best trade-off between hardware implementation and security. Finally, we perform an evaluation that determines how the proposed scheme can be used in an EPC Gen2 environment, since both statistical properties enforced by the standard and the power consumption needed for the available tags are inside the allowed boundaries. We take special care on power consumption measurement, performing an implementation of our scheme with the *LTspice IV* software [6], to better estimate energy requirements.

The paper is organized as follows. Section 2 surveys related work. Section 3 describes the design of J3Gen. Section 4 defines the optimal parameters used in the construction of J3Gen. Section 5 evaluates the statistical properties, hardware complexity and power consumption of J3Gen. Section 6 closes the paper.

## 2. Related Work

Although RFID is becoming an active research field in scientific literature, very few PRNG designs for lightweight RFID technologies have been disclosed in the related literature. Some examples are Trivium [7], Grain [8], and LAMED [2], all compatible with the EPC Gen2 requirements. Manufacturers of existing commercial solutions are, indeed, reluctant to provide their designs [2]. Moreover, some of the designs that do appear in the literature, and that claim to be both secure and lightweight enough to fit the EPC Gen2 restrictions, fail to provide convincing proofs of such claims. Some proper examples are [9,10]. The design in [9] is an optimized variant of the shrinking generator [11], a well-studied cryptographic design that combines two clocked linear feedback shift registers (LFSRs) [12]. The output sequence of the first LFSR is used to discard some bits from the output sequence of the second LFSR. However, it is worth pointing out that some techniques presented in [13] can be used to attack the scheme. Moreover, there are no evidences of how the proposal in [9] controls the irregularities of the generator output rate. This is an important drawback inherent to any shrinking generator scheme, since it can hint at the state of the main LFSR, and so breaking the security of the generator. The second example, presented in [10], is also a variant of the shrinking generator discussed above, but based on a physical source of randomness that handles the linearity of an underlying LFSR. In [14], Melià-Seguí *et al.* presented an efficient attack for successfully retrieving the feedback polynomial of this vulnerable generator scheme with very few observations. Assuming a 16-bit version of the generator, it was proved that the feedback polynomial can be predicted with a probability higher than 50% by simply capturing 160 bits, and 90% by capturing 464 bits. Therefore, the scheme does not meet any security standard.

The PRNG presented in this paper can be applied to current lightweight security proposals in wireless sensor networks, like the one-time-pad encryption scheme by Dolev *et al.* [15], the proactive threshold cryptosystem for EPC Tags by Garcia-Alfaro *et al.* [16], and the authentication protocols proposed by Delgado-Mohatar *et al.* [17], Liu and Peng [18], and Tounsi *et al.* [19].

It is worth to mention that there exist other PRNG implementations for security improvement like [20–22]. However, although efficient in their implementations, they cannot be applied to UHF technologies due to the technology state of the art, or power consumption criteria.

Considering EPC Gen2 RFID technology, Huang *et al.* proposed a PRNG-based authentication protocol specifically for EPC Gen2 [23]. Furthermore, the performance efficiency of the EPC Gen2 anti-collision protocol mainly depends on the on-board PRNG; hence, it is also of main importance [24]. Anti-collision improvement mechanisms such as the one presented by Mohsenian-Rad *et al.* [25] and Balachaldran *et al.* [26] are also based on the generation of pseudorandom sequences.

## 3. J3Gen Design

The main challenge to obtain an efficient PRNG is how to guarantee the generation of sequences with (almost) true random properties, while also addressing efficiency and computational complexity. Indeed, the low power, chip area and output rate (among other constraints) of EPC Gen2 technology makes the task of improving security harder. This is the case of true random number generator (TRNG) designs based on, e.g., thermal noise, high frequency sampling or fingerprinting, whose requirements of power consumption or computational complexity for full-length real-time generation of random sequences fall out of EPC Gen2 standards [1]. We propose to address this problem by combining a physical source of true randomness and a deterministic linear feedback shift register (LFSR) [14]. That is, leveraging the physical source system requirements with the efficiency of LFSRs for hardware implementations.

**Figure 1.**
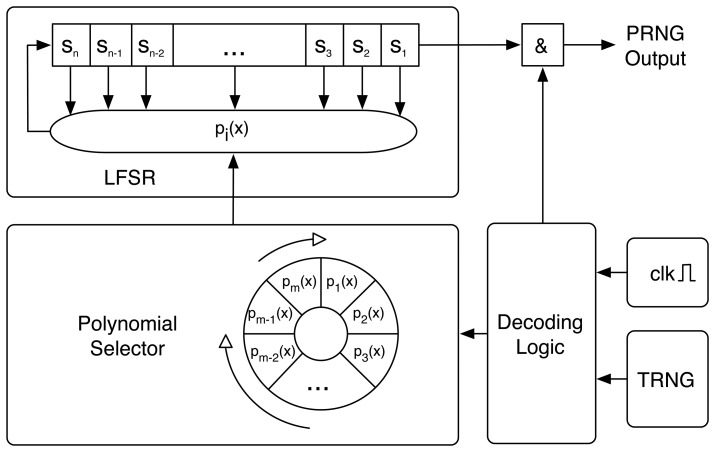
Block diagram of J3Gen.

Figure 1 depicts a block diagram of the J3Gen proposed design. It gets inspiration from a dynamic LFSR-based testing selection scheme presented by Hellebrand *et al.* in [27,28]. Indeed, it substitutes the static feedback polynomial configuration of an LFSR by a multiple feedback primitive polynomials configuration architecture. The different feedback primitive polynomials are connected to the LFSR by a decoding matrix that selects each single feedback polynomial. After a given number of LFSR cycles, the *Polynomial Selector Module* shifts its position towards a new configuration. The number of shifts, *i.e.*, the corresponding selection of each primitive polynomial at a certain LFSR cycle, is determined by a true random bit (hereinafter denoted as *trn*) that is obtained from a physical source of randomness. Next, the four main design modules are described. A detailed step by step sample execution of the scheme can be found in [5].

### 3.1. LFSR Module

The J3Gen generator relies on a n-cell LFSR module. LFSRs produce pseudorandom sequences with good statistical values. They are very fast and efficient in hardware implementations, and quite simple in terms of computational requirements [12]. This makes the use of LFSRs an ideal system for both energy and computational constrained environments. Moreover, LFSRs follow the same hardware scheme as those cyclic redundancy check (CRC) functions already included in the EPC Gen2 standard [1]. Therefore, current EPC Gen2 tags shall be able to execute LFSR-based functions in the same hardware.

### 3.2. Polynomial Selector Module

The Polynomial Selector is the responsible of the linearity avoidance of J3Gen. A set of *m* primitive feedback polynomials is selected, and each single feedback polynomial is used depending on the value of the truly random bit provided by the TRNG module. The feedback polynomials are implemented as a *wheel*, which rotates depending on the bit value given by the TRNG module. If the truly random bit is a logical 0, the *wheel* rotates one position, that is, it selects the next feedback polynomial. Instead, if the truly random bit is a logical 1, then the *wheel* rotates two positions, that is, the Polynomial Selector jumps one feedback polynomial and selects the next one.

### 3.3. Decoding Logic Module

The Decoding Logic is the responsible for managing the internal PRNG clock of J3Gen. It activates and deactivates the PRNG modules for its proper performance. The internal PRNG modules have different activation and deactivation timings. Depending on the internal clock frequency, *f*_clk_, some modules such as the LFSR or the TRNG need different activation cycles. For example, the *trn* sampling in the TRNG module is activated only once for each PRNG output.

The Decoding Logic also manages the *trn* obtained from the TRNG module, rotating the Polynomial Selector with regard to the *trn* value. This action is performed using *l* cycles, with 1 ≤ *l* ≤ *n* − 1. This value is lower than the *n* cycles needed in the LFSR to avoid pseudorandom sequences generated from a single feedback polynomial. In this way, the generated sequence does not have linearity properties and common attacks to retrieve the equivalent LFSR generator, like the Berlekamp–Massey algorithm, are not able to perform.

### 3.4. Thermal-Noise TRNG

Regarding the physical source of randomness (*trn*), there are different proposals to derive true random sequences of bits from the hardware of a radio-frequency identification (RFID) tag. The technique used in J3Gen is the oscillator-based high frequency sampler by Che *et al.* [10], which offers high simplicity and suitability for EPC Gen2 designs. To leverage the high power consumption of this technique for EPC Gen2 standards [10,14], the TRNG output is optimized with the LFSR Module described in Section 3.1, which divides the TRNG operating frequency by *n* as described in Section 3.3. The output of the TRNG is fed to the Decoding Logic which, in turn, manages the Polynomial Selector.

## 4. Parameters Set-Up

Although J3Gen can be used as a security tool in multiple lightweight ubiquitous computing scenarios, we look for compatibility with the EPC Gen2 requirements. The EPC standard [1] does not define any hardware requirement for the generation of the 16-bit pseudorandom sequences, nor any other hardware security features regardless of the CRC that shares the LFSR scheme with our proposal (*cf.* Section 3). Authors in the literature do not agree about the implementation area that can be devoted to security. Using logical gates equivalence (GE) to measure the implementation size (regardless of the manufacturing process), some authors defend that only 2000 GE can be used for security [29,30], while others increase the range from 2000 to 5000 [31]. In this paper, the worst case is assumed. Hence, J3Gen looks for a suitable trade-off between security and hardware implementation cost, that is, maximizing the security inside the proposed implementation area of 2000 GE. This implementation area is approximately 10, 400 *μ*m^2^ using current 130 nm process for static CMOS designs.

### 4.1. Hardware

The size and design of each component of J3Gen implies a specific hardware implementation, being the LFSR size (*n*) and the number of implemented feedback polynomials on tag (*m*) the parameters that most significantly impact on the hardware complexity of our design. Furthermore, both parameters are also the key parameters from the security point of view, hence, we shall look for the different combinations of *n* and *m* within the hardware implementation boundaries to find the best security implementation for this purpose.

Table 1 represents the different implementation counts (based on GE area), depending on the main PRNG parameters *n* and *m*, as described in Section 3.3. The *n*-bit register is included in the LFSR module, the LFSR feedback and decoder are included in the Polynomial Selector module, and the cycle clock, TRNG and PRNG output are included in the Decoding Logic module. These three elements add up to the most representative amount of GEs. The remaining GEs mainly consist of the necessary extra circuitry for controlling the different states of the generator. Logic gates considered in this implementation count are basic two-input gates, except for the *decoder* (Polynomial Selector) where (*log*(*m*)*/log*(2))*-input* NAND gates are used depending on the number of implemented polynomials *m*.

For the LFSR implementation purpose, we use the *D-flip-flop* (DFF) model specified at [32] composed by 18 CMOS transistors. Hence, a DFF can be measured with approximately 4.5 GE.

As shown in Table 1, the Polynomial selector module implementation hardly depends on the total number of polynomials *m* that will be used as a pool of feedback polynomials. Furthermore, the exact selection of the *m* polynomials also affects the total number of GE needed for the implementation. To determine the GE number for this module, a first approach could be to analyze all combinations of the *m* possible polynomials and take the combination that needs the minimum GE. Although this strategy seems to be the best one regarding the implementation purposes for its efficiency (it will end up with the simplest implementation possible), it is not a good choice regarding security needs. As we will describe in the next section, the exact value of the *m* polynomials (not the value *m* itself) can be seen as a security key (since, together with the *trn* values, it determines the output of the pseudorandom sequence). Then, an attacker could determine the exact combination of the used polynomials simply by finding the combinations that produce the optimal implementation in GE. To avoid that, we compute the GE needed in this module by analyzing all the possible polynomial combinations and then take the worst possible case. Since any chosen combination of the *m* polynomials is equally probable, an attacker cannot discard any of them regarding its implementation suitability. Based on this strategy, we show in Table 1 the resulting GE upper bound values.

We then provide the physical source of randomness assumed for our generator. For the gate equivalence of this component, we based our estimations on previous works presented in [31,33]. The physical source of randomness that we assume consists of the thermal-noise oscillator presented by Che *et al.* in [10], but specified and modeled in our work as proposed in [34,35].

From Table 1, it can be extracted that implementations using up to 32 cells for the LFSR are roughly EPC Gen2 suitable from the hardware perspective. Also, a combination of large LFSR with a small pool of polynomials (e.g., *n* = 64 and *m* = 8) offers a possible solution, regarding hardware constrains (bold values in the Total row). In the next subsection, we overview the security properties of our scheme for those parameters that fit the hardware constrains, discarding implementations surpassing the available implementation area, established in 2000 GE.

### 4.2. Security

EPC Gen2 security relies on the PRNG utilization, and how the PRNG ciphers the *Access* or *Kill* operations [1] to avoid eavesdroppers to obtain the cleartext of the transmitted sequences. That is, the security of an EPC Gen2 PRNG is based on the unpredictability of its output. In J3Gen, such unpredictability is based on the non-linearity module that depends on the combination of the selected *m* feedback polynomials, and the feedback polynomial update rate *l*. In our scenario, assuming that *n*, the length of the LFSR, is a public value, the knowledge of the exact combination of *m* feedback polynomials would allow to an attacker to predict the output sequence. In that context, since such polynomials are kept secret, they may be considered as the secret key of our PRNG. Then, the security strength of J3Gen can be measured as a key length, understanding each key as every different possible *m* feedback polynomial combination.

In order to achieve the best statistical properties, feedback polynomials of LFSRs shall be primitive [12]. Table 2 shows, for each LFSR length *n*, the total number of existing primitive polynomials. Given such value, each possible number of *m* chosen feedback polynomials determines the total available combinations that can be taken to implement our scheme. Each combination represents a possible key, so the powers of the values of the fourth column (labeled as *Possible combin*.) in Table 2 determines the length of the key (in bits). At this point, it is worth mentioning that different authors point out that a security of 80 bits is adequate for low-cost RFID [33,36], then all our combinations (except the first one) provide sufficient key length.

**Figure 2.**
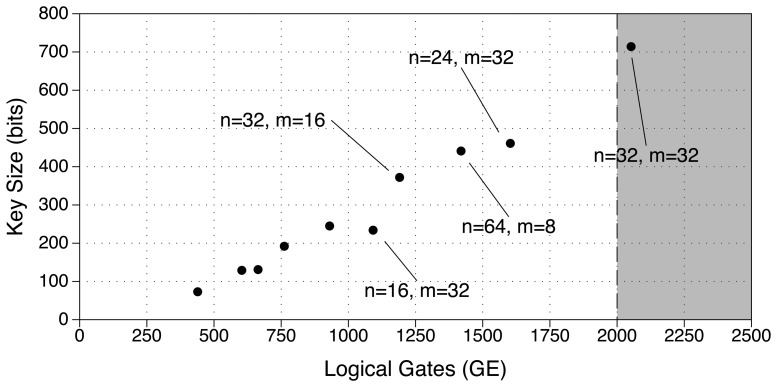
Different combinations present suitable trade-offs between security and implementation area.

Figure 2 depicts the equivalent-key size regarding the necessary logic GE for the implementation of J3Gen. An implementation with *n* = 32 and *m* = 32 slightly exceeds the 2000 available GE for security purposes. Using less feedback polynomials reduces the implementation area in exchange for smaller key sizes. This is the case of combining *n* = 32 and *m* = 16, which can be implemented with 1191 logic GE giving a key of 372 bits. Figure 2 shows that, although GE and key bit length grows in parallel, such relation is not uniform, since with 98 GE we can increase 138 bits in key length when moving from the implementation with *n* = 16 and *m* = 32 to the parameters *n* = 32 and *m* = 16, while 184 GE are required to increase only 20 key bits from *n* = 64 and *m* = 8 to *n* = 24 and *m* = 32.

For that reason, we determine the best parameters to choose in terms of GE efficiency with respect to the key length. Taking that measure, we obtain that the best parameter configuration is *n* = 32 and *m* = 16, where the ratio between the key length and GE is maximized (last column of Table 2). That is, using an LFSR register with 32 cells (*n* = 32) and 16 feedback polynomials (*m* = 16) implemented on the tag.

Regardless of the parameters values, the core of the J3Gen generator is an LFSR with multiple polynomials (instead of a single one). The polynomial generator from a simple *n*-cell LFSR with period 2*^n^* − 1 can be determined with only 2*n* bits due to the linearity of this method, by using a system of *n* equations or the Berlekamp–Massey algorithm [37]. The linearity of the J3Gen is avoided with the following technique. The parameter *l* described in Section 3.3 avoids the generation of more than *l* consecutive bits from each LFSR polynomial. Depending on the level of desired security, *l* can be bounded by 1 ≤ *l* ≤ *n* − 1. Moreover, each *n*-bits pseudorandom sequence is generated by at least two different polynomials.

An attacker aiming to predict the J3Gen output has to synchronize its output with the beginning of a feedback polynomial generated sequence, obtain the feedback polynomial from 2*n* using the method described above, and repeat the same operation for each *m* feedback polynomials. Here, the attacker has to face with the uncertainty added by the feedback update rate (*l*). For example, using the selected parameters *n* = 32 and *m* = 16, if *l* = 31 it means there would be up to 4 possible solutions for each system of equations, that is, up to 4 possible feedback polynomials generating that sequence. If *l* = 25 then the possible solutions are up to 16,384, for *l* = 21 the possible solutions increase to 4,194,304, *etc.* The extreme case would be *l* = 1 where all 67 million primitive feedback polynomials would be equally probable (*cf.*Table 2). Thus, the smaller the polynomial update cycle *l*, the harder the attack because more bits would be needed to disclose all *m* feedback polynomials. For instance, a *l* = 31 needs about 1400 bits to obtain all primitive polynomials, *l* = 25 needs about 134,000 bits, and *l* = 21 needs about 33 million bits. Hence, depending on the desired level of security, the attack will need a longer output sequence of consecutive bits. Equation (1) bounds the probability of success of each attack to 2*n* bits, where *p_i_*(*x*) are the obtained polynomials and *P*_sel_ are the *m* implemented polynomials on the generator.

(1) $$\frac{1}{2^{n-l+1}}\leq P(p_{i}(x)\in P_{\text{sel}})\leq 1$$

If further security is desired, the pool of polynomials can include non-primitive polynomials besides primitive polynomials (avoiding those leading to absorbing states), increasing the complexity of the system and decreasing the success odds of a brute force attack.

## 5. EPC Gen2 Suitability

Once the parameters have been fixed based on the hardware constraints and the security requirements discussed in previous sections, we now evaluate the proposed scheme for its implementation, and the restrictions imposed by the EPC standard. We analyze two important parameters of J3Gen: statistical requirements stated by the EPC Gen2 standard for pseudorandom sequence generation, and power consumption.

### 5.1. Statistical Performance

Detailed in the EPC Gen2 standard [1], the requirements for the pseudorandom sequence generation can be summarized as follows:

1. Any single 16-bit sequence *s* drawn from the generator shall confirm Equation (2).
  (2) $${\text{P}}_{\min}=\frac{0.8}{2^{16}}<\text{prob}(S\text{)}<{\text{P}}_{\max}=\frac{1.25}{2^{16}}$$
2. Among a tag population of up to ten thousand tags, the probability that any two tags simultaneously generate the same 16-bit sequence shall be less than 0.1%.
3. The chance of guessing the next 16-bit sequence generated by a tag shall be less than 0.025% even if all previous outputs are known to an adversary.

To confirm the suitability of the current design of J3Gen for handling the statistical and randomness requirements defined above, different pseudorandom sequences using the parameters defined in Section 4 shall be generated. The three EPC Gen2 statistical requirements tests are presented. To accomplish these tests, 30 million 16-bit pseudorandom sequences are generated using an implementation of the J3Gen design. This dataset size is chosen since it is the minimum necessary for a truly generated random sequence to accomplish the proposed requirement [5].

**Figure 3.**
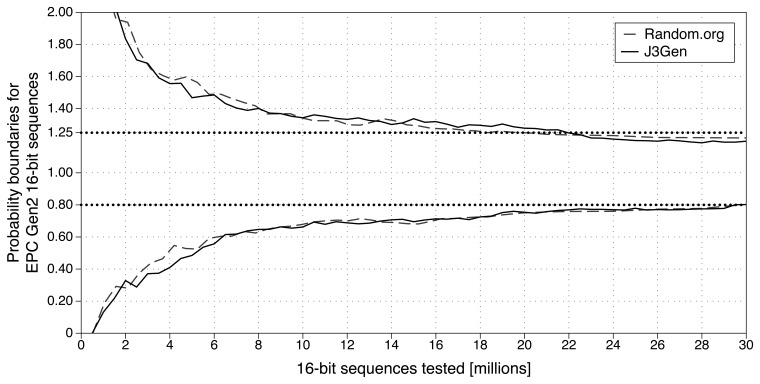
EPC Gen2 first randomness property test, achieving similar statistical results than *Random.org* true random sequences.

First, the probability of occurrence of any given value shall lie between probabilities defined in Equation 2. The results shown in Figure 3 confirm that, after analyzing 30 million 16-bit sequences, the probability of occurrence of any given value lies between the defined boundaries. Furthermore, J3Gen achieves similar statistical results with *Random.org* true random sequences [38], based on its frequency properties.

The second property for building an EPC Gen2 compliant PRNG requires that two simultaneous identical sequences must not appear with more than 0.1% probability for a population up to 10,000 tags. To test this property, 10,000 instances of J3Gen, initialized at random, are used to simulate the test scenario. We conducted ten tests, running 1,000 iterations per test. The second row of Table 3 (labeled as *Same* 16*-bit sequence*) presents the obtained results. We can observe that all the tests show a simultaneous identical sequence rate one order of magnitude smaller than requested by the standard.

Finally, to statistically confirm the fulfillment of the third property, we computed the degree of dependence of the ongoing bits regarding their predecessors, using the same pseudorandom sequences. Based on the results shown in Table 3, we can confirm that the generated sequences are not predictable within the requested correlation of 0.025%.

### 5.2. Power Consumption

The energy used for a (cryptographic) operation depends on the average power and the duration of the computation. For passively powered devices such as RFID tags, the average power transmitted from the reader to the tag is relatively small (although, in general terms, the reader can supply the power during all the operation time [39]). Standard CMOS transistors is the current choice of most digital circuit designs built for low power consumption and robustness [31]. Feldhofer *et al*. have estimated the average power budget for cryptographic operations in 4 *μ*W at five meters to the reader [39]. Therefore, it is important for the implementation of J3Gen to not surpass the available power budget.

Analytical methods for estimating the CMOS dynamic power dissipation can be adapted to the design of J3Gen [31]. Indeed, using custom values adapted to the J3Gen design, the average power consumption is estimated in P_avg_ = 178 nW (readers can refer to [5] for details).

After defining the design of the digital core of J3Gen based on GEs (*cf.* Section 4), we conduct an electronic circuit simulation of the proposed J3Gen construction, using very-large scale integration (VLSI). The simulation language SPICE [40] is used to simulate the circuit, and the *LTSpice IV* software [6] is used to represent the circuit using logical gates. The resulting simulation also allows us to demonstrate the fundamental concepts of the current construction of J3Gen and confirm its validity as a stand-alone device.

Power dissipation is one of the most important factors in VLSI design and its technology choice. Therefore, accurate simulation of CMOS power dissipation using languages such as SPICE is highly desirable [41]. To precisely evaluate the power consumption of our design, it is necessary to provide libraries with parameter models of the specific technology that is simulated. These libraries include a variety of CMOS parameters modeling the transistor behavior and parasitic circuit elements. Using library models, which can be theoretically modeled or hardware measured [40], the precision of the calculations is improved to effectively simulate the circuit like a real fabricated device. Current UHF RFID products are fabricated on 180 and 130 nm CMOS processes [39]. For our simulation we use the *Predictive Technology Model* (PTM) libraries [42] provided by the Nanoscale Integration and Modeling Group from the Arizona State University, which provides CMOS models for 130 nm processes.

**Figure 4.**
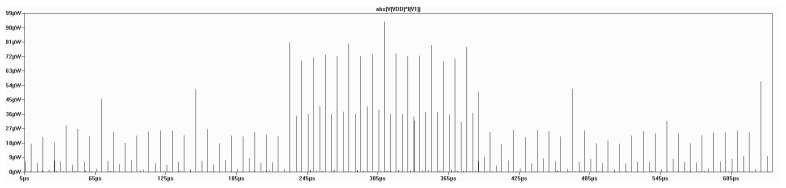
LTSpice power consumption simulation. Power dissipation is concentrated around the internal clock cycles.

The analysis targets the average power consumption of J3Gen, in order to evaluate its implementability in a real EPC Gen2 tag. Figure 4 depicts the PRNG power consumption during one 16-bit sequence, generated with LTSpice IV and the PTM libraries.

The simulated average power consumption for the 16-bit sequence generation is 156.3 nW, which is consistent with the aforementioned dynamic CMOS power estimation. The simulated power consumption is also under the average power consumption requirements for cryptographic operations in RFID tags proposed by Feldhofer *et al.* [39].

## 6. Conclusions

A pseudorandom number generator (PRNG) design for EPC Gen2 RFID technologies, named J3Gen, has been presented. The generator is based on a linear feedback shift register (LFSR) configured with a multiple-polynomial tap architecture fed by a physical source of randomness, achieving a reduced computational complexity and low-power consumption as required by the EPC Gen2 standard. It is intended for security, improving the one-time-pad cipher unpredictability. It is configurable for other purposes and scenarios besides EPC Gen2 RFID technologies through its main parameters *n* (LFSR size) and *m* (number of polynomials). The proposed architecture results in a security equivalent-key size of 372 bits, in opposition to the linearity of a single LFSR generator. We have validated the nonlinearity of the design and its suitability with regard to the EPC Gen2 standard. Specifically, we have considered the randomness requirements through a statistical analysis, the hardware complexity with a logical gates design, and the power consumption through an evaluation based on CMOS parameters and SPICE language simulation. Besides EPC Gen2 compatibility, stronger security can be obtained by increasing the main parameters of J3Gen.

## Figures and Tables

**Table 1.** Logical GE Count for J3Gen.

LFSR size (*n*)	16			24			32			64		

Feedback polynomials (*m*)	8	16	32	8	16	32	8	16	32	8	16	32
LFSR module	72.0	72.0	72.0	108.0	108.0	108.0	144.0	144.0	144.0	288.0	288.0	288.0
Polynomial Selector module	209.3	396.6	774.1	305.1	577.6	1,125.3	401.0	758.6	1,476.5	784.3	1,482.4	2,881.3
Decoding Logic module	48.3	48.3	48.3	53.3	53.3	53.3	53.3	53.3	53.3	61.3	61.3	61.3
TRNG	22.0	22.0	22.0	22.0	22.0	22.0	22.0	22.0	22.0	22.0	22.0	22.0
Additional Control	87.5	125.0	182.1	114.9	169.3	279.9	141.2	212.7	356.3	249.3	387.9	667.7

**TOTAL**	**439.1**	**663.9**	**1092.5**	**603.3**	**930.2**	**1,602.8**	**761.5**	**1,190.6**	2,052.1	**1,419.3**	2,241.6	3,920.3

**Table 2.** Combinations using primitive polynomials.

**LFSR size (*n*)**	**Primitive polynomials**	**Num. of pol. (***m*)	**Possible combin.**	**Gate Eq. (GE)**	**Ratio (bit/GE)**
		8	2^73^	439.1	0.1662
16	2,048	16	2^131^	663.9	0.1973
		32	2^234^	1092.5	0.2141

		8	2^129^	603.3	0.2138
24	276,480	16	2^245^	930.2	0.2633
		32	2^461^	1,602.8	0.2876

32	67,108,864	8	2^192^	761.5	0.2521
		16	2^372^	1,190.6	**0.3124**

64	1.44 ×10^17^	8	2^441^	1,419.3	0.3107

**Table 3.** EPC Gen2 second and third randomness property tests.

**Test (%rate)**	1*st*	2*nd*	3*rd*	4*th*	5*th*	6*th*	7*th*	8*th*	9*th*	10*th*
**Same 16-bit sequence**	0.0377	0.0383	0.0377	0.0370	0.0375	0.0375	0.0369	0.0375	0.0371	0.0379
**Correlation**	–0.0085	–0.0093	–0.0044	–0.0014	0.0003	0.0053	0.0073	0.0038	–0.0020	–0.0178
